# Poly(2-methyl-2-oxazoline) as a polyethylene glycol alternative for lipid nanoparticle formulation

**DOI:** 10.3389/fddev.2024.1383038

**Published:** 2024-04-26

**Authors:** Dwain George van Zyl, Livia Palmerston Mendes, Raphaela Patricia Semper, Christine Rueckert, Patrick Baumhof

**Affiliations:** CureVac SE, Tübingen, Germany

**Keywords:** lipid nanoparticle (LNP), polyethelene glycol (PEG), poly(2-methyl-2-oxazoline) PMOZ, mRNA delivery, vaccine

## Abstract

Lipid nanoparticles (LNPs) have emerged as the platform of choice for mRNA delivery. Polyethylene glycol (PEG) is considered a key component of currently approved LNP-based delivery systems as it ensures particle stability and shapes various facets of LNP behavior in biological systems. Whilst PEG has numerous characteristics that are favorable for delivery systems, there is a growing body of evidence that suggests that it is immunogenic. Thus, next-generation mRNA therapeutics are likely to benefit from the identification of PEG alternatives. Towards this end, we have assessed the suitability of poly(2-methyl-2-oxazoline) (PMOZ) for LNP-based mRNA delivery. We compared the properties and bioactivities of PMOZ-containing LNPs to that of a standard composition that includes PEG. Decreasing the percentage of PMOZ in formulations improved transfection efficiency and enhanced the immunostimulatory potential. Reducing the PMOZ density was shown to enhanced antigen-specific T-cell responses *in vivo*. Interestingly, we found that this was not the case for antibody responses. A direct comparison between LNPs that contain the same amount of PEG or PMOZ strongly suggests that the former induces stronger CD8^+^ T-cell responses while the latter induces superior neutralizing titers. These findings augur well for the further development of PMOZ as a PEG replacement for LNP-based mRNA delivery approaches.

## 1 Introduction

Lipid nanoparticles (LNPs) are an emerging platform for mRNA delivery. Encapsulation of mRNA in LNPs protects from nucleases and enables delivery to the cytosol of cells where mRNA can be recognized by the cellular translation machinery (Geall et al., 2012). LNPs traditionally comprise four lipid types the ionizable aminolipid, cholesterol, phospholipid, and polyethylene glycol (PEG)-lipid. The ionizable aminolipid is considered one of the most important LNP constituents as it enables mRNA encapsulation, promotes endosomal escape and contributes to the immunostimulatory profile of LNPs (Semple et al., 2010; Alameh et al., 2021). Cholesterol and phospholipid contribute to the structure and stability of particles (Cheng and Lee, 2016; Kawaguchi et al., 2023). The PEG-lipid serves as a stabilizer in aqueous solutions and accordingly increases colloidal and biological stability (Bao et al., 2013). However, the amount of PEG-lipid can impact a myriad of LNP properties that include encapsulation efficiency, particle size, zeta potential and transfection efficiency (Belliveau et al., 2012; Bao et al., 2013; Kumar et al., 2014).

PEG is the most widely used polymer for drug and vaccine delivery purposes. Conjugation of PEG bestows pharmaceuticals with so-called stealth properties which increases circulation half-lives, reduce protein binding, and ultimately improves efficacy (Wang et al., 2023). Additionally, PEG is non-toxic and positively influences the safety profile of pharmaceuticals (Turecek et al., 2016). The biocompatibility of PEG has also been the reason for its inclusion in a wide array of consumer products. However, the popularity of PEG set the stage for an increasing number of studies that found PEG to display immunogenicity in humans (Armstrong et al., 2007; Fang et al., 2021; Estapé Senti et al., 2022; Ju et al., 2022). Anti-PEG antibodies can be induced by treatment with PEG-based biopharmaceuticals or may even be generated in untreated, healthy individuals through environmental exposure. These findings underscore the need to identify alternatives to PEG for use in delivery systems such as LNPs.

Considering the limitations associated with PEG based polymers, it is not surprising that the delivery field has attempted to identify PEG alternatives. Numerous synthetic polymers and polypeptoids have been investigated and include poly(2-oxazolines) (POZ) (Woodle et al., 1994), polyglycerol (Abu Lila et al., 2013), polysarcosine (Nogueira et al., 2020; Wilhelmy et al., 2023) and poly(N-methyl-N-vinylacetamide (Berger et al., 2023). The POZ family contains two notable members with short side chains poly(2-ethyl-2-oxazoline) (PEOZ) and poly(2-methyl-2-oxazoline) (PMOZ) that are fully water-soluble. These members display low cytotoxicity, are biocompatible (Kronek et al., 2011) and have promising antifouling properties (Najer et al., 2022). These valuable properties are the reason PEOZ and PMOZ have been explored as conjugates for nanoformulations (Woodle et al., 1994; Qiu et al., 2013; Zhang et al., 2017; Wan et al., 2019). However, these polymers are chemically distinct from PEG and have different physical properties (Estapé Senti et al., 2022; Wilhelmy et al., 2023), which imply careful optimization may be required to achieve comparable performance when applied to LNP-based mRNA delivery.

Here, we present a proof of concept for the replacement of the PEG-lipid by an alternative for the purpose of prophylactic vaccination. We compare PMOZ-containing LNPs to that of a standard PEG-based formulation in terms of physicochemical properties, stability, and functionality in various biological models.

## 2 Materials and methods

### 2.1 mRNA design and synthesis

All mRNAs used consisted of CAP-carrying linear mRNA with a 5′untranslated region (UTR) from the human hydroxysteroid 17-beta dehydrogenase 4 gene (HSD17B4), a 3’ UTR from the human proteasome 20S subunit beta 3 gene (PSMB3) and a histone stem-loop followed by an A100 poly(A) tail (Gebre et al., 2022), either encoding for Rabies virus glycoprotein G (RABV-G) (N1-methylpseudouridine (m1Ψ)-modified, RABV-G mRNA) or encoding for enhanced green fluorescent protein (unmodified, eGFP mRNA). Furthermore, all open reading frames were GC-optimized (Thess et al., 2015). All mRNAs were produced in-house (CureVac SE) (Tübingen, Germany).

### 2.2 Formulation of LNPs

**FIGURE 1:**
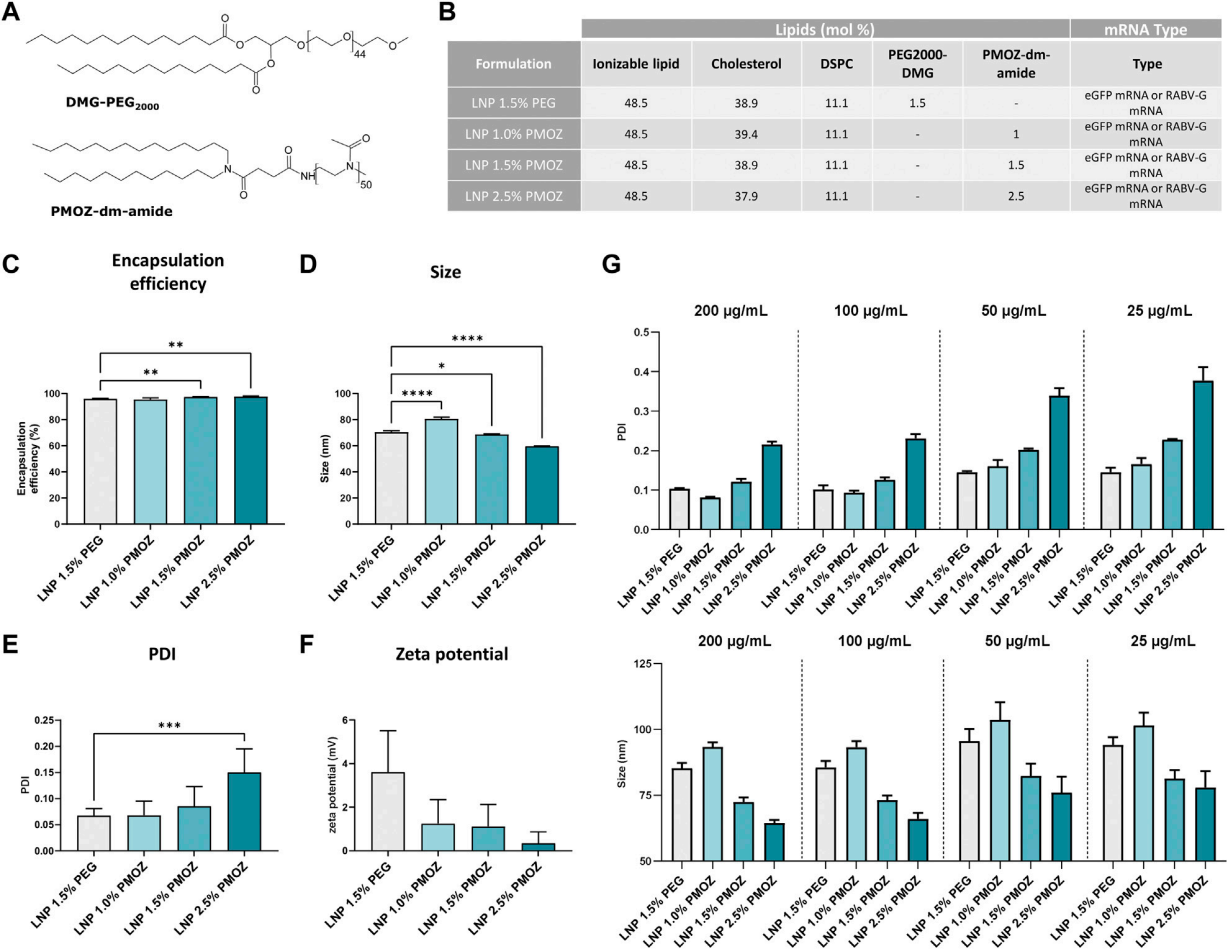
Physicochemical characteristics of LNPs formulated with various percentages of PMOZ. **(A)** Chemical structure of PEG2000-DMG and PMOZ-DM-amide. **(B)** Composition of mRNA-LNPs that contain different polymer-lipid percentages. Formulation was performed with either unmodified eGFP mRNA or m1Ψ-modified RABV-G mRNA. **(C)** Encapsulation efficiency (%) of mRNA. **(D–F)** DLS analysis of hydrodynamic size **(D)**, PDI **(E)** and zeta potential **(F)**. Triplicate measurements were performed for LNPs that encapsulate unmodified eGFP mRNA or m1Ψ-modified RABV-G mRNA and bars represent mean values. **(G)** RABV-G mRNA-LNPs were frozen at various concentrations and PDI (top panel) and hydrodynamic size (bottom panel) analyzed upon thawing. Triplicate measurements are shown and bars represent mean values. Statistical analysis was performed with a one-way ANOVA with Tukey’s multiple comparison test. *p < 0.1, **p < 0.01, ***p < 0.001, ****p < 0.0001.

Lipid Nanoparticles (LNPs) were prepared using the microfluidics mixing technique, where an ethanolic lipid solution is mixed with an aqueous mRNA solution. The lipid solution contained ionizable lipid H (Broadpharma, USA), cholesterol (Avanti Polar Lipids, USA), Dioctadecanoyl-sn-glycero-3-phosphocholin (DSPC) (Avanti Polar Lipids, USA), and 1,2-Dimyristoyl-rac-glycero-3-methylpolyoxyethylene glycol-2000 (DMG-PEG2000) (NOF Corporation, Japan) (mol ratio 48.5:38.9:11.1:1.5) or N-methyl-2-(N4', N4′-di(tetradecyl)succinamide)-poly[(N-acetyl)ethylamine] (PMOz-DM-amide) (4778.20 g/mol, purity >95% (LC-UV); CureVac) replacing PEG at various mol% (1.0%, 1.5% and 2.5%), all dissolved in ethanol, while the mRNA was diluted in 50 mM citrate (pH 4.0) (see more details in Figure 1). The device used for the mixing was the Nanoassemblr^®^ Ignite™ (Precision Nanosystems, Vancouver, Canada) and the solutions were mixed at a volume ratio of 3:1 (mRNA solution:lipid solution) and at a flow rate of 20 mL/min. After mixing, the LNPs were transferred to dialysis cassettes (Slyde-a-Lyzer G3) (ThermoFisher, USA) and dialyzed overnight, with 2 buffer exchanges in between, against a physiological buffer. After dialysis, the LNPs were concentrated using Vivaspin^®^ ultrafiltration units (Satorius, Germany) and physicochemical features were characterized. Hydrodynamic diameter size and polydispersity index (PDI) were measured by dynamic light scattering using the DynaPro Plate Reader III (Wyatt, USA). The zeta potential was measured with a Zetasizer Nano ZS90 (Malvern, United Kingdom). mRNA concentration and encapsulation efficiency were determined via Quant-iT Ribogreen^®^ RNA assay (ThermoFisher, USA) following the manufacturer’s recommendations. Briefly, the mRNA is diluted with TE buffer in the presence or absence of Triton X-100 and the Ribogreen^®^ dye was added to the samples, followed by their measurement at 485 nm excitation and 535 nm emission. Samples treated with Triton X-100 represent the total mRNA (LNPs are broken down in the presence of the detergent) and the samples in absence of Triton X-100 comprise “external” mRNA. The encapsulation efficiency is defined by $EE\;\left(\%\right)=\frac{\text{RNA}\left(\text{total}\right)-\text{RNA}\left(\text{external}\right)}{\text{RNA}\left(\text{total}\right)}∙100\%$ . The composition and physicochemical properties of all LNPs are summarized in Figure 1.

### 2.3 Cell lines and primary cells

Cell lines used in this study include HeLa (DSMZ ACC57), HepG2 (DSMZ ACC 180), 293T (ATCC CRL-3126) and THP-1 Null2 (InvivoGen) cells. Blood was purchased from the Zentrum für Klinische Transfusionsmedizin (Tübingen, Germany) and human peripheral blood mononuclear cells (PBMCs) were isolated with Ficoll-Paque PLUS (Cytiva) densitiy gradient medium and cryopreserved in 90% FCS and 10% DMSO until required. HeLa, HepG2 and PBMCs were maintained in RPMI (brand/specification; supplemented with 10% FCS, 2 mM L-Glutamine, 100 μg/mL Pen-Strep), while 293T cells were cultured in DMEM (brand/specification; supplemented with 10% FCS, 2 mM L-Glutamine, 100 μg/mL Pen-Strep). THP-1 Null2 cells were maintained in RPMI (brand/specification; supplemented with 10% FCS, 25 mM HEPES, 100 μg/mL Pen-Strep, 100 μg/mL Normocin, 100 μg/mL Zeocin). All cells were cultured at 37°C, 5% CO_2_.

### 2.4 LNP transfection of cell lines *in vitro*

HeLa, 293T and HepG2 cells were respectively seeded overnight (37°C, 5% CO_2_) in 6-well plates at 2.5 × 10^5^ cells/well, 3.0 × 10^5^ cells/well and 5.0 × 10^5^ cells/well, and THP-1 Null2 cells in 24-well plates at 5.0 × 10^5^ cells/well. LNP-formulated eGFP mRNA was added to the culture medium to a final concentration of 10 ng/mL (for transfection of HeLa, 293T and HepG2 cells) and 500 ng/mL (for transfection of THP-1 Null2 cells), respectively. Cells were incubated for another 18 h at 37°C, 5% CO_2_, stained with Fixable Viability Stain 780 (BD Biosciences) and the eGFP translation product was detected via flow cytometry. Data were respectively acquired and analyzed with the NovoCyte Penteon flow cytometer (Agilent Technologies) and FlowJo software (v10.8.1).

### 2.5 LNP transfection of PBMCs *ex vivo*

PBMCs were seeded at 1.5 × 10^6^ cells in 24-well plates and treated with LNPs at 500 ng/mL or control (medium) for 18 h at 37°C, 5% CO_2_. Supernatants were collected, cleared by centrifugation and chemokines and cytokines were quantified (see section 2.6). The cells were stained with Fixable Viability Stain 780 (BD Biosciences, #565388), anti-HLA-DR (R718, clone L243, BD Biosciences, #568578), anti-CD14 (BUV395, clone MφP9, BD Biosciences, #563561), anti-CD3 (BUV496, clone SK7), anti-CD19 (BUV563, clone SJ25C1, BD Biosciences, # 612916), anti-CD45 (BUV805, clone HI30, BD Biosciences, #612891), anti-CD11c (BV480, clone B-ly6, BD Biosciences, #566135), anti-CD16 (BV786, clone 3G8, BD Biosciences,# 563690), anti-CD56 (PE-Cy7, clone B159, BD Biosciences, #557747) and anti-CD123 (PE, clone 7G3, BD Biosciences, # 568155) to identify different immune cell populations. Staining was additionally performed with anti-CD80 (BV605, L307.4, BD Biosciences, #563315), anti-CD83 (PE-Cy5, clone HB15e, BD Biosciences, #551058), anti-CD86 (BV421, clone 2331 (FUN-1), BD Biosciences, # 562432) to identify activated antigen presenting cells. The fixable viability dye and antibodies were obtained from BD Biosciences. Data were respectively acquired and analyzed with the NovoCyte Penteon flow cytometer (Agilent Technologies) and FlowJo software (v10.8.1).

### 2.6 Quantification of cytokines and chemokines

LEGENDplex multi-analyte Flow assays (Biolegend) were used to quantify cytokines and chemokines in the present study. Cell culture supernatants were collected after 18 h (at 37°C, 5% CO_2_) of treatment with 500 ng/mL of LNPs. The resulting supernatants were analyzed with the Human Anti-Virus Response Panel (13-plex) and enabled quantification of IL-1β, IL-6, CXCL8 (IL-8), IL-10, IL-12p70, IFN-α2, IFN-β, IFN-λ1 (IL-29), IFN-λ2/3 (IL-28a/b), IFN-γ, TNF-α, CXCL10 (IP-10) and GM-CSF. For *in vivo* studies, sera were collected 14 h after primary immunization and analyzed with the Mouse Anti-Virus Response Panel (13-plex) and enabled quantification of IFN-γ, CXCL1 (KC), TNF-α, CCL2 (MCP-1), IL-12p70, CCL5 (RANTES), IL-1β, CXCL10 (IP-10), GM-CSF, IL-10, IFN-β, IFN-α and IL-6.

### 2.7 Quantification of RABV-G-specific T cells

Spleens were harvested on day 28 and single cell suspensions prepared. The resulting cells were stained with Zombie UV Fixable Viability dye (Biolegend, #423108), α-CD90.2 (AF700, 53-2.1, Biolegend, #140324), α-CD8a (BUV737, clone 53-6.7, BD Biosciences, #612759), α-CD4 (BV786, clone RM4-5, BD Biosciences, #563727) and a class I (H-2Ld) pentamer (APC, ProImmune Ltd.) loaded with 453-461aa of RABV-G. Data were respectively acquired and analyzed with the NovoCyte Penteon flow cytometer (Agilent Technologies) and FlowJo software (v10.8.1).

### 2.8 Animal studies

Animal experiments were performed at Synovo GmbH (Tübingen, Germany). Female BALB/cJRj mice 8–9 weeks old were randomly divided into five groups (n = 7) and injected intramuscularly (*Musculus tibialis*) with 25 µL of mRNA LNP formulation (1 µg of mRNA per dose) or buffer control at days 0 (primary immunization) and 21 (secondary immunization). Blood was collected 14 h after the primary immunization, allowed to clot and serum prepared by centrifugation and the resulting material was used for chemokine and cytokine determination (see section 2.6). Serum was prepared in a similar manner on day 28 for quantification of virus neutralizing titers (VNTs) by EUROVIR Hygiene-Labor GmbH (Luckenwalde, Germany). Splenocytes from day 28 were analyzed for the presence of antigen-specific CD8^+^ T-cell responses (see section 2.7).

### 2.9 Measurement of AST and ALT liver

Mice were injected i.m. with 25 µL of LNP 1.5% PEG and LNP 1.5% PMOZ (1 µg of mRNA per dose) or buffer and blood was collected after 14 h. The blood was allowed to clot and sera were prepared via centrifugation. The resulting sera were diluted 1:10 in 0.9% NaCl solution and AST/ALT levels were quantified with the response^®^910VET Veterinary Chemistry Analyzer (DiaSys Diagnostic Systems, USA) according to the manufacturers’ instructions.

### 2.10 Statistical analysis

Statistical analysis was performed using GraphPad Prism software (version 10.1.0). Data were assessed by a one-way ANOVA with Tukey’s multiple comparison test or by Brown-Forsythe and Welch ANOVA with Dunnett’s T3 multiple comparison tests. Only statistically significant data are illustrated on figures and significant differences are defined as *p < 0.1, **p < 0.01, ***p < 0.001, ****p < 0.0001.

## 3 Results

### 3.1 Characterization of LNPs that contain PMOZ

To study PMOZ as a PEG alternative we selected SM-102 LNP (Jackson et al., 2020) as a model system. Towards this end, SM-102 LNP (hereafter simply referred to as LNP) was formulated with ionizable lipid H (Hassett et al., 2021), DSPC, cholesterol and polymer-lipid (Hald et al., 2022). The polymer-lipids used in this case were either PEG2000-DMG or PMOZ-DM-amide. Both contain a C14 lipid anchor, while PEG2000-DMG or PMOZ-DM-amide respectively comprise polymer repeating units of 44 or 50 (Figure 1A). PEG2000-DMG was formulated at 1.5 mol% and the resulting LNP served as reference material for the present study. PMOZ-DM-amide was formulated at 1.0 mol%, 1.5 mol% or 2.5 mol% and the amount of cholesterol was adjusted to achieve a total of 100 mol% for all lipid components of the LNP (Figure 1B). LNPs were prepared with either unmodified eGFP mRNA or N^1^-methylpseudouridine (m1Ψ)-modified RABV-G mRNA. Ribogreen assay analysis revealed that replacement of PEG by PMOZ does not influence mRNA encapsulation efficiency. All LNPs consistently showed encapsulation efficiencies over 95% (Figure 1C). Next, we assessed the physicochemical characteristics of the LNPs via dynamic light scattering (DLS) and this showed that the percentage of PMOZ influenced the hydrodynamic size (Figure 1D), polydispersity index (PDI) (Figure 1E) and zeta potential (Figure 1F) of the LNPs. Higher percentages of PMOZ correlated with decreases in hydrodynamic size and zeta potential. Conversely, the percentages of PMOZ correlated negatively with the PDI. Next, we evaluated how dilution influences changes in hydrodynamic size and PDI in response to freeze-thaw (Figure 1G). These data show that PMOZ-containing LNPs have physicochemical characteristics that are typical for LNPs used for mRNA delivery for medical applications and raise no significant concerns for further testing in biological models.

### 3.2 Testing the functionality of LNPs *in vitro* and *ex vivo*

**FIGURE 2:**
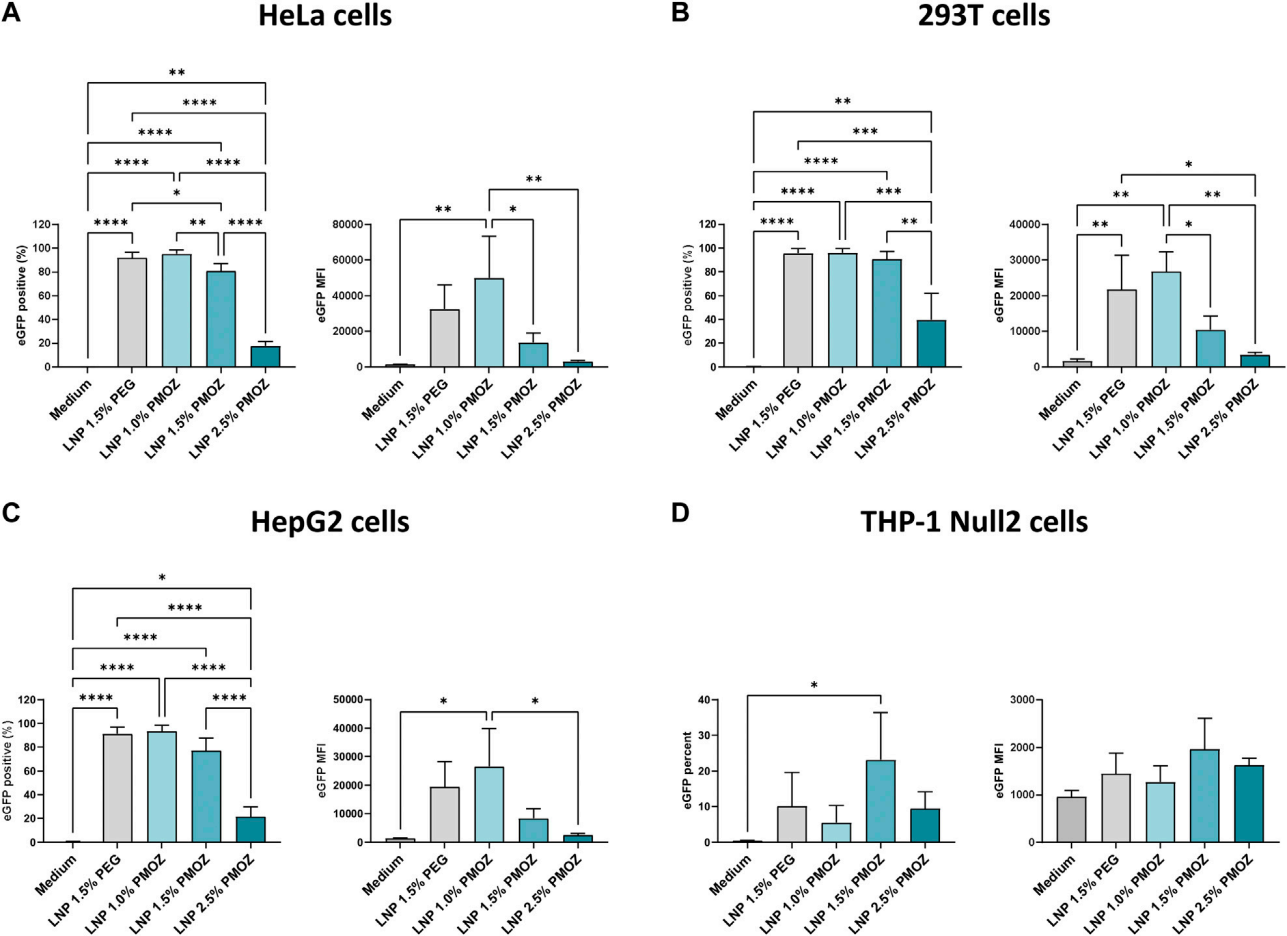
Transfection of various cell lines with eGFP mRNA-LNPs that comprise different PMOZ percentages. **(A–D)** HeLa **(A)**, 293T **(B)** and HepG2 **(C)** cell lines were transfected with 10 ng/mL of LNPs, while the THP-1 Null-2 **(D)** cell line was transfected with 500 ng/mL of LNPs. Medium served as the negative control. The degree of transfection was determined as percentage eGFP-positive cells (left panel) and eGFP MFI (right panel). Bars represent the mean of three independent experiments and errors bars represent standard deviation. Statistical analysis was performed with a one-way ANOVA with Tukey’s multiple comparison test. *p < 0.1, **p < 0.01, ***p < 0.001, ****p < 0.0001.

We next assessed the performance of PMOZ-containing LNPs in various cell lines. To this end, HeLa, 293T, HepG2 and THP-1 Null2 cells were treated with LNPs that encapsulate unmodified eGFP mRNA. The resulting cells were analyzed by flow cytometry to quantify the percentage eGFP-positive cells and the eGFP median fluorescence intensity (MFI) (Figure 2; Supplementary Figure S1). This revealed that a lower percentage of PMOZ correlated with an increase in transfection in HeLa, 293T and HepG2 cells. Moreover, LNP 1.0% PMOZ (for LNP nomenclature see Figure 1B) transfected these cell lines to a similar degree as LNP 1.5% PEG. This trend was not observed in the monocytic cell lines THP-1 Null2 and LNP 1.5% PMOZ more readily transfected cells in comparison to all other LNPs. However, this trend is not statistically significant. These results strongly suggest that LNPs can be formulated with PMOZ to successfully deliver mRNA to cell lines.

**FIGURE 3:**
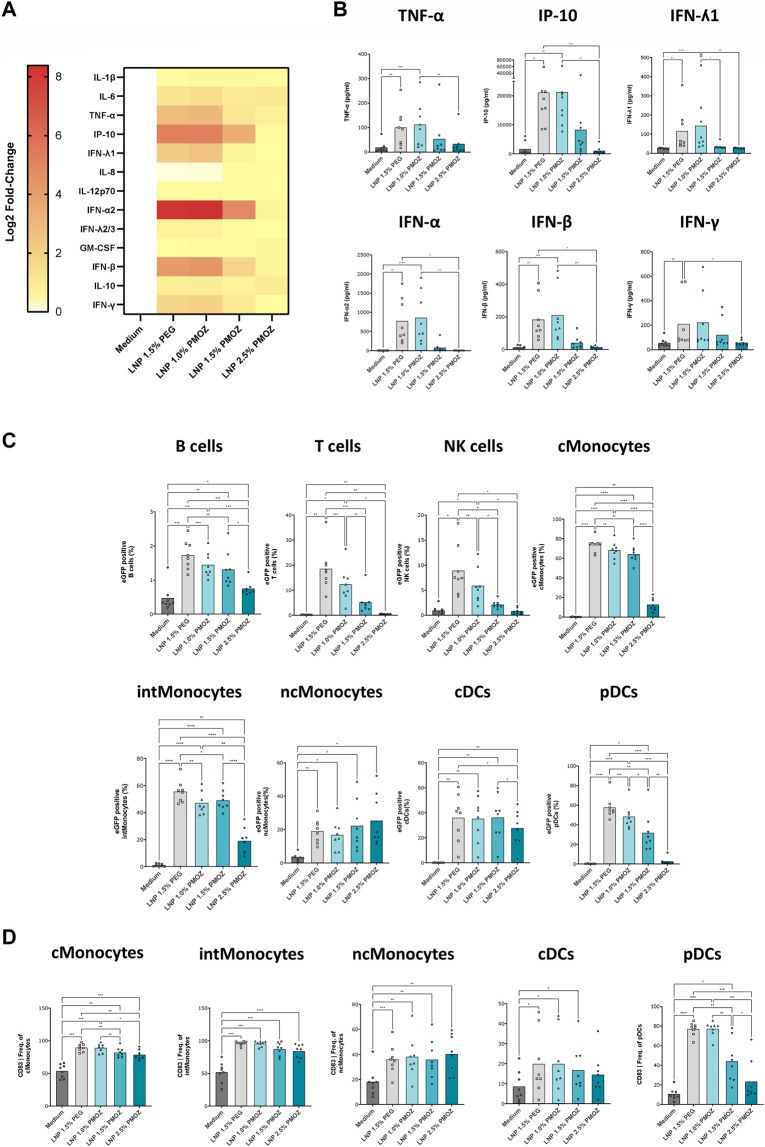
Analysis of eGFP mRNA-LNPs that comprise different percentages of PMOZ *ex vivo*. PBMCs from 8 donors were treated with LNPs at 500 ng/mL or medium control for 18 h. **(A)** Heatmap of Log2-transformed fold-change values (relative to medium) for various chemokines and cytokines. **(B)** Quantification data are shown for selected chemokine and cytokines. **(C)** The degree of transfection was determined in various immune cell populations using flow cytometry. The percentage of eGFP-positive cells was determined. **(D)** Activated innate immune cells were identified through increased CD83 surface expression. Percentages are given and bars represent the mean of 8 donors. Statistical analysis was performed with a Brown-Forsythe and Welch ANOVA with Dunnett’s T3 multiple comparison test. *p < 0.1, **p < 0.01, ***p < 0.001, ****p < 0.0001.

Having confirmed that PMOZ-containing LNPs successfully transfected cell lines, we aimed to test them in primary immune cells. Accordingly, we selected PBMCs as they comprise numerous cell types and enable the immunostimulatory properties of LNPs to be determined (Tahtinen et al., 2022). PBMCs from several donors were treated overnight with eGFP mRNA LNPs and the resulting supernatants and cells were analyzed (Figure 3). The immunostimulatory profile of the LNPs was determined by quantifying cytokines and chemokines in cell culture supernatants using a cytokine bead array (Figures 3A,B). This revealed that a select number of cytokines was induced by the tested LNPs (Figure 3A). Quantification data from TNF-α, IP-10, IFN-ʎ1, IFN-α, IFN-β and IFN-γ show that LNP 1.5% PEG and LNP 1.0% PMOZ were potent immune activators. In contrast LNP 1.5% PMOZ and LNP 2.5% PMOZ less readily induced cytokines and there is a general trend that suggests increasing PMOZ percentages have a negative impact on immune stimulation. Next, we performed detailed analysis on the various adaptive and innate immune cell populations that are transfected by LNPs that contain PEG or PMOZ lipid-polymers (Figure 3C; Supplementary Figure S2, S3). LNP 1.5% PEG most readily transfected the majority of immune cell types in comparison to LNP 1.0% PMOZ, LNP 1.5% PMOZ and LNP 2.5% PMOZ. The only observed exception are non-classical monocytes, where the type and percentage of polymer-lipid did not have a significant impact on the percentage of cells that contain the eGFP translation product. Lastly, we determined the degree of innate cell activation by quantifying the percentage of cells that express the costimulatory molecules CD80, CD83 or CD86 at their surface (Figure 3D; Supplementary Figure S4). This showed that there is a clear relationship between the amount of PMOZ and immunostimulatory potential, with increasing PMOZ percentages corresponding with decreased chemokine and cytokines values. Moreover, it is evident that LNP 1.5% PEG and LNP 1.0% PMOZ displayed a similar phenotype, and most readily induced activation in the majority of innate cells. These data demonstrate that the *in vitro*/*ex vivo* transfection efficiency and immunostimulatory potential of LNPs can be modulated by changing the amount of PMOZ.

### 3.3 Immunization of mice with LNPs that encapsulate RABV-G mRNA

**FIGURE 4:**
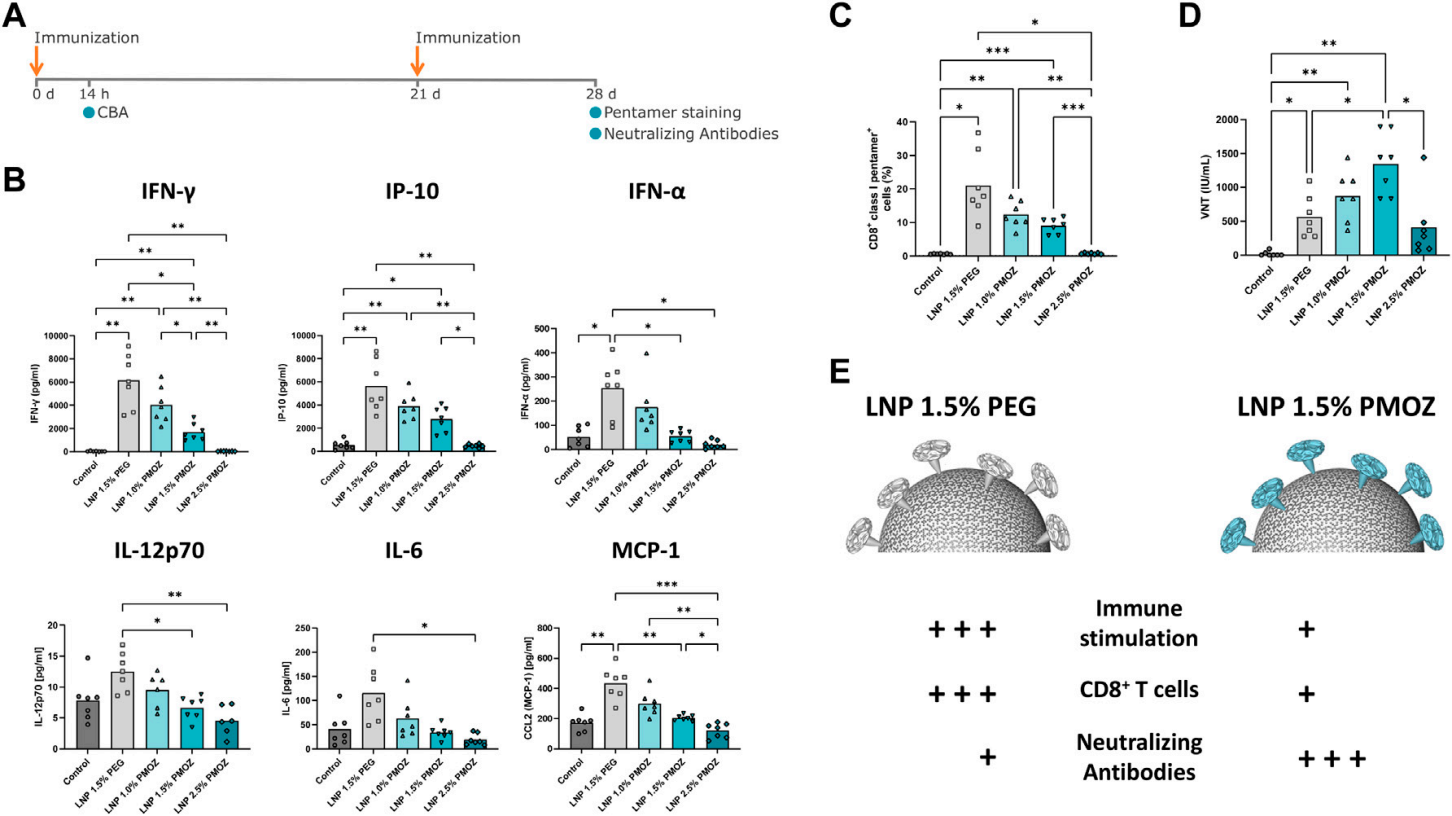
Vaccination of mice with LNPs that contain mRNA encoding the Rabies virus glycoprotein (RABV-G). **(A)** Mice were injected **(I)** m. with 1 µg of RABV-G mRNA-LNPs at days 0 and 21. The immunization schedule is displayed along with the various tests that were performed. **(B)** Cytokine levels in the sera of animals were quantified using a cytokine bead array. **(C)** Antigen specific CD8^+^ T-cell responses in spleens sampled on day 28. Splenocytes were stained with CD8-specific antibody and RABV-G class I pentamer to identify CD8^+^ class I pentamer^+^ cells. **(D)** Virus neutralization titers (VNTs) were quantified in sera from day 28. Statistical analysis was performed with a Brown-Forsythe and Welch ANOVA with Dunnett’s T3 multiple comparison test. Bars represent the mean of 7 animals. *p < 0.1, **p < 0.01, ***p < 0.001, ****p < 0.0001. **(E)** Differential immune responses are induced by PEG and PMOZ LNP.s.

To interrogate LNPs containing PMOZ in a vaccination setting, LNP-encapsulated m1Ψ-modified RABV-G mRNA was used to immunize BALB/cJRj mice. Animals were injected intramuscularly on day 0 and day 21 with a dose of 1 µg mRNA (Figure 4A). The immunostimulatory profiles of the LNPs were determined 14 h after the primary immunization by quantifying various chemokines and cytokines in the sera of animals (Figure 4B). LNP 1.5% PEG most robustly induced cytokine responses, while the tendency of PMOZ-containing LNPs to induce cytokines *in vivo* increased as the percentage of PMOZ was decreased. Next, we assessed RABV-G-specific CD8^+^ T-cell responses present in animals at day 28 (Figure 4C; Supplementary Figure S5). To this end, splenocytes were stained with a class I pentamer loaded with the RABV-G epitope LPNWGKYVL (453-461 aa). The percentage of double-positive CD8^+^class I pentamer^+^ cells were clearly higher in animals immunized with LNP 1.5% PEG relative to LNPs that contain PMOZ (1.0%, 1.5% or 2.5%). However, this observation is only statistically significant when comparing LNP 1.5% PEG and LNP 2.5% PMOZ. Lastly, sera from day 28 were analyzed for the presence of rabies virus neutralizing antibody titers (VNTs) (Figure 4D). This showed that all animals vaccinated with m1Ψ-modified RABV-G mRNA LNPs, irrespective of lipid-polymer type and percentage, had titers that exceed the minimal protection threshold (≥0.5 IU/mL) (World Health Organization, 2018). However, notable differences can be observed between treatment groups. Our results show that LNP 1.5% PMOZ induced the highest mean VNTs, a result that is statistically significant when compared with LNP 1.5% PEG. Interestingly, LNP 1.0% PMOZ also induced higher mean VNTs than LNP 1.5% PEG, although this finding is not statistically significant. Together these findings suggest that LNPs formulated with PMOZ (1 mol% and 1.5 mol%) give rise to a superior VNT response, but less readily induce antigen-specific CD8^+^ T-cell responses when compared to LNPs formulated with PEG.

## 4 Discussion

Although LNP-based delivery has found tremendous success in the form of SARS-CoV-2 vaccines (Polack et al., 2020; Baden et al., 2021), there is interest to apply this technology to a plethora of indications for prophylactic and therapeutic purposes (Hou et al., 2021). The identification of novel lipid components with unique properties is likely to facilitate the development of next-generation LNPs. There is a growing body of evidence that suggests that the PEG-lipid is immunogenic (Ibrahim et al., 2022) and indicates the identification of PEG alternatives will benefit LNP-based delivery systems. Along these lines, it is desirable to identify an alternative moiety with the same functionality that lacks the detrimental effects associated with the use of PEG-lipid. Here we report on a PEG alternative, PMOZ, that shows potential to be used in LNPs for mRNA vaccine delivery. We tested this in the background of an mRNA-LNP formulation that reflects the design principles of the SARS-CoV-2 Spikevax vaccine (Baden et al., 2021). We compared the properties and bioactivities of a standard PEG-containing composition with modified versions that comprise different concentrations of PMOZ.

The ability of LNPs to deliver their nucleic acid payload to the cytosol of cells is a critical parameter for assessing efficacy. We opted for transfection-based approaches with LNPs that encapsulate reporter eGFP mRNA and analyzed their transfection efficiency in various continuous cell lines and primary immune cells. This showed that LNPs formulated with lower percentages of PMOZ transfected cell lines and immune cells at a higher rate (1.0% > 1.5% > 2.5). Of these, LNP 1.0% PMOZ achieved transfection efficiencies in the same range as LNP 1.5% PEG. The observation that lower percentages of PMOZ correlates with increased transfection rates, agrees with previous work that studied PEG-containing LNPs (Bao et al., 2013; Kumar et al., 2014; Patel et al., 2023). This suggests that previously described relationship between polymer-lipid percentage and transfection efficiency holds true for PMOZ polymers. The polymers are suggested to limit membrane fusion within endocytic vesicles by providing a steric barrier (Kumar et al., 2014). This could explain why LNPs with lower polymer percentages display higher transfection efficiencies.

The steric barrier afforded by the polymer-lipid also has ramifications for LNPs *in vivo*. In addition to affecting transfection (Ryals et al., 2020), the steric barrier impacts the degree to which LNPs interact with biomolecules. Higher PEG percentages limit the formation of the biomolecular corona at the surface of the LNPs and this can influence endogenous targeting mechanisms after intravenous application (Chen et al., 2016; Dilliard and Siegwart, 2023). Accordingly, PEG endows drugs with so-called stealth properties so that they are not as efficiently recognized by the mononuclear phagocyte system (MPS), ultimately extending the circulation half-life of drugs (Ekladious et al., 2019). Thus, LNPs with lower percentages of polymer-lipid are more likely to be cleared by the MPS upon intravenous administration (Shan et al., 2009; Lee et al., 2023). PMOZ-containing liposomes were previously shown to display similar plasma clearance kinetics as PEG-containing liposomes in mice (Zalipsky et al., 1996), suggesting that differential clearance of PEG- and PMOZ-containing LNPs by the MPS is not expected to play a significant role in the present study. However, it is difficult to interpret this comparison in context of the human population, since PMOZ is currently not as widely used as PEG, with the latter being a common additive in cosmetics, food and medicine (Ibrahim et al., 2022), and anti-PEG IgM and IgG are commonly detected in the sera of healthy individuals (Estapé Senti et al., 2022). As opposed to PMOZ-containing LNPs, PEG-containing LNPs might thus display enhanced clearance by the MPS system in a subset of the human population (Estapé Senti et al., 2022). However, a definitive conclusion on such matters will only be possible, and relevant, once the human population is exposed to PMOZ-containing products. Moreover, since most studies that deal with MPS clearance of LNPs focus heavily on i.v. application, it remains to be determined to what extent these findings apply to prophylactic vaccination via i.m. injecton. A deeper understanding polymer-lipid stealth properties in i.v. and i.m. settings will inform design of LNPs for local and systemic delivery.

Reactogenicity is another important factor that may be mediated or modulated by polymer-lipid. The reactogenicity displayed by approved SARS-CoV-2 mRNA-LNP vaccines is hypothesized to be mediated, in part, by anti-PEG antibodies. Several studies have analyzed PEG-specific antibodies in individuals vaccinated with approved LNP-based SARS-CoV-2 vaccines Spikevax and Comirnaty (Carreño et al., 2022; Ju et al., 2022; Kozma et al., 2023). These studies showed that PEG-specific IgG and IgM are more readily induced after boost and that titers are more notable in individuals who received Spikevax than Comirnaty. The analysis of a small cohort of patients revealed that increased levels of PEG-specific IgG and IgM are associated with an increased incidence of systemic reactogenicity (Ju et al., 2022). These data suggest that LNP-based vaccines that contain PEG may directly contribute to reactogenicity but further studies are required to conclude whether this is the case, and if so, to what extent (Ju et al., 2023). Along these lines, PMOZ is not widely used and is still in its infancy as a polymer in medical products, so there are currently no data available on anti-PMOZ antibodies in the human population. To our knowledge, a POZ drug-conjugate has only been tested in humans in a single instance and this involves PEOZ (Moreadith et al., 2017). The drug in question was found to be safe, well tolerated and did not induce any adverse events (Olanow et al., 2020). It remains to be determined whether this holds true for PMOZ-based delivery systems, but considering the high structural similarity between PEOZ and PMOZ, these findings Bode well for the continued development of LNPs that contain PMOZ. Polymer-lipid is also thought to contribute to reactogenicity of mRNA-LNPs by modulating their immunostimulatory profile. Increasing amounts of PEG have been shown to blunt LNP-mediated cytokine production *in vitro* and *in vivo* (Kumar et al., 2014). This observation is confirmed by our data which show that chemokine and cytokine levels decreased *ex vivo* and *in vivo* upon treatment with LNPs that contained increasing amounts of PMOZ. A direct comparison of LNP 1.5% PEG and LNP 1.5% PMOZ, strongly suggests that the immunostimulatory potential of the latter is significantly lower.

Immunization experiments in mice showed that LNPs formulated with 1.0% and 1.5% PMOZ induced notable T- and B-cell responses. However, it appears that these LNPs did not induce antigen-specific T-cell responses to the same degree as LNPs that contain 1.5% PEG. Strikingly, the inverse was found when VNTs were assessed and suggests that the aforementioned PMOZ-containing LNPs induce superior antibody responses to that of the LNP with 1.5% PEG. It is difficult to speculate at this time about the reasons that underline these observations. However, the LNPs in the present study differed in size. In accordance with previous studies performed with PEG (Semple et al., 2010; Belliveau et al., 2012; Lokugamage et al., 2021), we found that PMOZ percentages negatively correlated with size. Whilst we have not determined biodistribution in the present study, it is known that particle size can influence biodistribution, with non-LNP particles less than 200 nm in size freely draining to lymph nodes (Manolova et al., 2008; Bachmann and Jennings, 2010; Hassett et al., 2021). A specific analysis of LNPs of different sizes (98 nm, 181 nm or 327 nm) indicated that LNPs of 98 nm drained more efficiently from the injection site when compared to larger LNPs (Di et al., 2022). Since the LNPs within the present study were much smaller and within a narrow size range (60–80 nm), it is difficult to delineate whether size might have impacted drainage to relevant lymphoid organs. Another study examined LNPs within the 60–200 nm range and found that LNPs between 85 nm and 100 nm in size induced optimal antibody responses (Hassett et al., 2021). If these observations hold true for the LNPs in the current study, it suggests that LNP 1.0% PMOZ with the largest size of 80 nm would be the optimal size for induction of humoral immune responses. However, this is not the case since LNP 1.5% PMOZ with an approximate size of 69 nm induced the highest VNTs. Interestingly, LNP 1.5% PEG had a similar size (70 nm) and induced dramatically lower VNTs when compared to LNP 1.5% PMOZ. This suggests that LNP size alone is unlikely to account for the differences observed between the LNPs we have studied. We also analyzed AST and ALT liver enzymes in animals vaccinated with LNP 1.5% PEG and LNP 1.5% PMOZ (Supplementary Figure S6), and our data show no significant difference between these two groups. This suggest that differential liver toxicity did not contribute to differences between LNP 1.5% PEG and LNP 1.5% PMOZ in our vaccination study. Lastly, the lack of biodistribution information is an obvious limitation of the present study and such data are likely to provide further insights into the mode of action characteristics of LNPs that contain PMOZ.

Polymer-lipid endows LNPs with specific physicochemical properties and governs various aspects of their behavior. Thus, it is not surprising that changing the nature and/or abundance of polymer-lipid can have far-reaching implications for the behavior of LNPs in biological systems and exemplifies the challenges associated with finding an alternative to PEG. We demonstrate that PEG lipids can be replaced by PMOZ lipids in functional LNPs for mRNA delivery in a vaccination setting. We show that LNP characteristics and functions such as particle size, zeta potential, PDI (Figure 1), *in vitro* cell transfection and immune stimulation (Figure 2; 3) and immunogenicity (Figure 4) can be modulated by using different PMOZ-lipid concentrations in the mRNA LNP formulation. We conclude that the PMOZ-lipid may be expanded to applications beyond the model system we explored here, for example, to different mRNA-encoded antigens, different LNP compositions, long-term immunity readouts, therapeutic (cancer) vaccine or other multi-dose (molecular therapy) applications that rely on systemic delivery.

## Data Availability

The raw data supporting the conclusion of this article will be made available by the authors, without undue reservation.

## Supplementary material

SUPPLEMENTARY FIGURE S1 Viability of cells treated with LNPs. Cells were stained. Statistical analysis was performed with a one-way ANOVA with Tukey’s multiple comparison test. *P < 0.1, **P < 0.01, ***P <0.001, ****P < 0.0001.

SUPPLEMENTARY FIGURE S2 Gating strategy for the analysis of adaptive and innate cells. PBMCs were treated with LNPs or medium and were then cultured for 18 hours. The resulting cells were analysed by flow cytometry to identify B cells, T cells, NK cells, classical Monocytes (cMonos), intermediate Monocytes (intMonos), non-classical Monocytes (ncMonos), conventional Dendritic cells (cDCs) and plasmacytoid Dendritic cells (pDCs). Transfection efficiency (eGFP) or activation (CD80, CD83, CD86) were respectively determined for all cell types or innate immune cells.

SUPPLEMENTARY FIGURE S3 Viability of PBMCs treated with LNPs. Statistical analysis was performed with a one-way ANOVA with Tukey’s multiple comparison test. *P < 0.1, **P < 0.01, ***P <0.001, ****P < 0.0001.

SUPPLEMENTARY FIGURE S4 Analysis on innate immune cell activation through the surface expression of activation markers CD80 **(A)** and CD86 **(B)**. Percentages are given and bars represent the mean of 8 donors. Statistical analysis was performed with a Brown-Forsythe and Welch ANOVA with Dunnett’s T3 multiple comparison test. *P < 0.1, **P < 0.01, ***P <0.001, ****P < 0.0001.

SUPPLEMENTARY FIGURE S5 Analysis of antigen-specific CD8^+^ T cells in mouse spleens on day 28. The gating strategy employed **(A)** and representative contour plots of CD8^+^ class I pentamer^+^ cells **(B)** are shown.

SUPPLEMENTARY FIGURE S6 ALT and AST levels in the sera of animals treated with buffer (control), LNP 1.5% PEG and LNP 1.5% PMOZ. Sera were collected with 14 hours after the primary vaccination. Statistical analysis was performed with a one-way ANOVA with Tukey’s multiple comparison test. *P < 0.1, **P < 0.01, ***P <0.001, ****P < 0.0001.
