# Supplementary material for: Poly(2-methyl-2-oxazoline) as a polyethylene glycol alternative for lipid nanoparticle formulation
Source: Front Drug Deliv. 2024 Apr 26;4:1383038. doi: 10.3389/fddev.2024.1383038 (PMC12363279; doi:10.3389/fddev.2024.1383038)
Supplement: Supplementary file 1 [file DataSheet1.docx]

# Supplementary Material


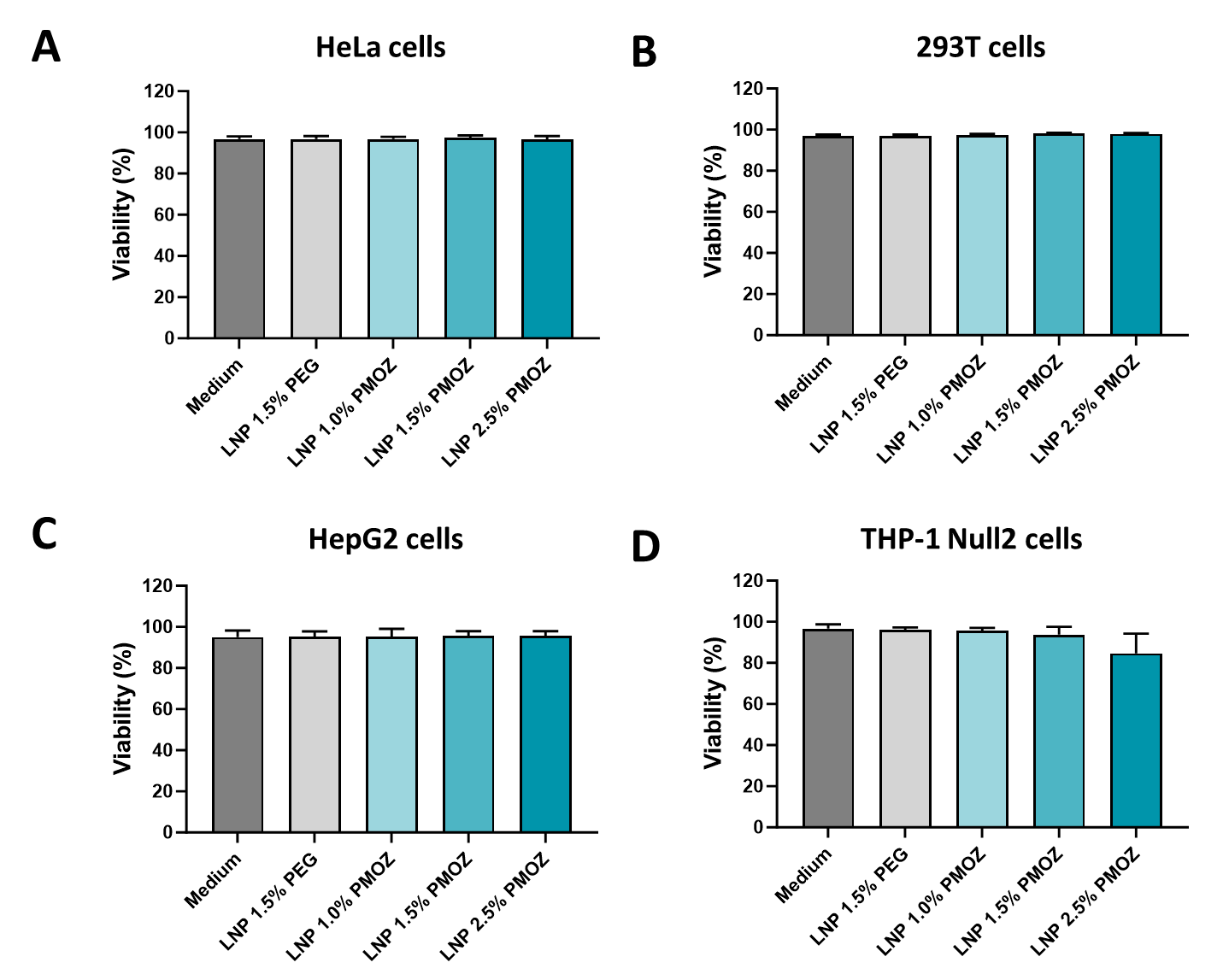


Supplementary Figure 1: Viability of cells treated with LNPs that encapsulate eGFP mRNA. Dead cells (FVS780 dye-positive) were identified by flow cytometry. Statistical analysis was performed with a one-way ANOVA with Tukey’s multiple comparison test. *P < 0.1, **P < 0.01, ***P <0.001, ****P < 0.0001.

**
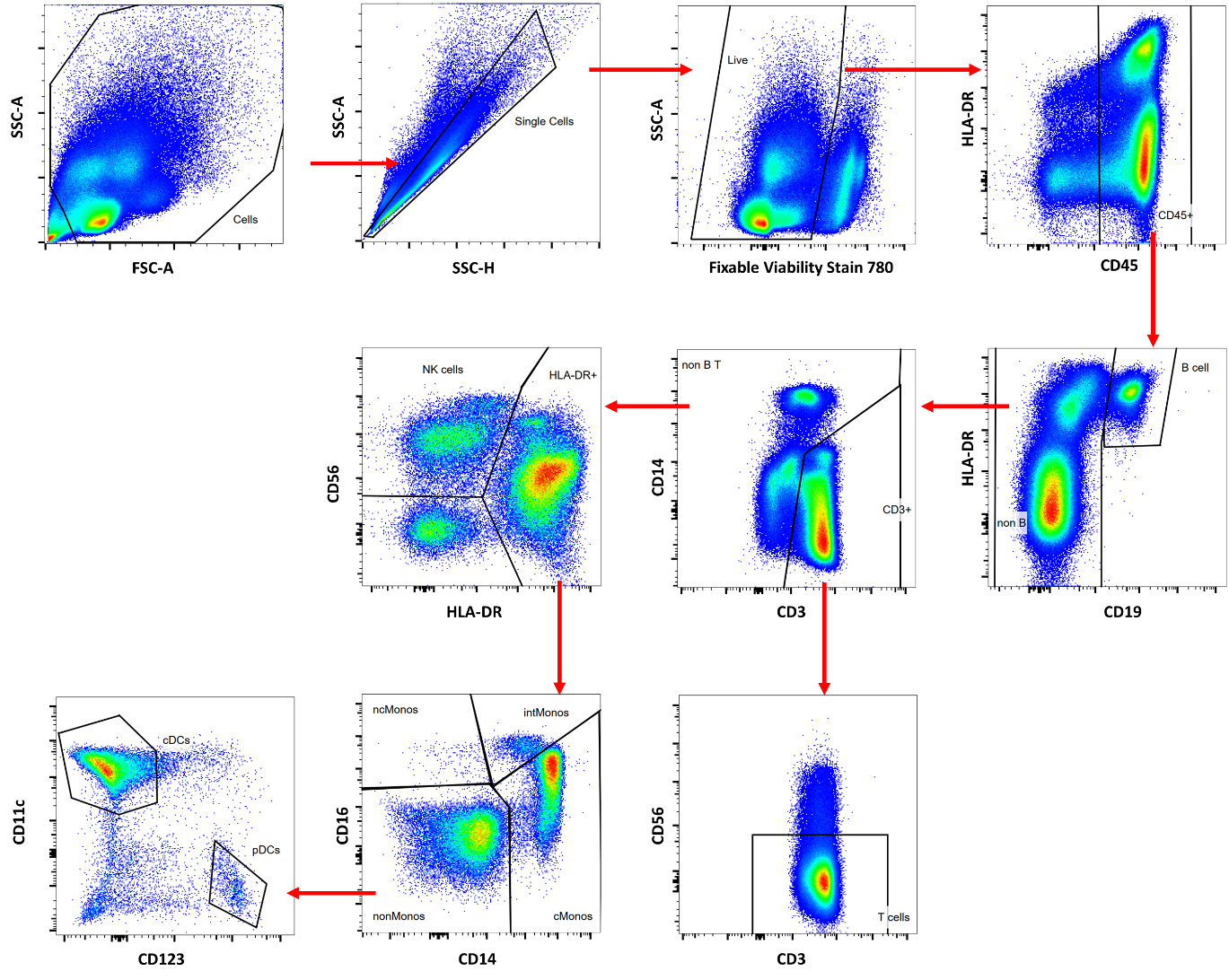
**

Supplementary Figure 2: Gating strategy for the analysis of adaptive and innate cells. PBMCs were treated with medium or LNPs that encapsulate eGFP mRNA and were then cultured for 18 hours. The resulting cells were analysed by flow cytometry to identify B cells, T cells, NK cells, classical Monocytes (cMonos), intermediate Monocytes (intMonos), non-classical Monocytes (ncMonos), conventional Dendritic cells (cDCs) and plasmacytoid Dendritic cells (pDCs). Transfection efficiency (eGFP) or activation (CD80, CD83, CD86) were respectively determined for all cell types or innate immune cells.


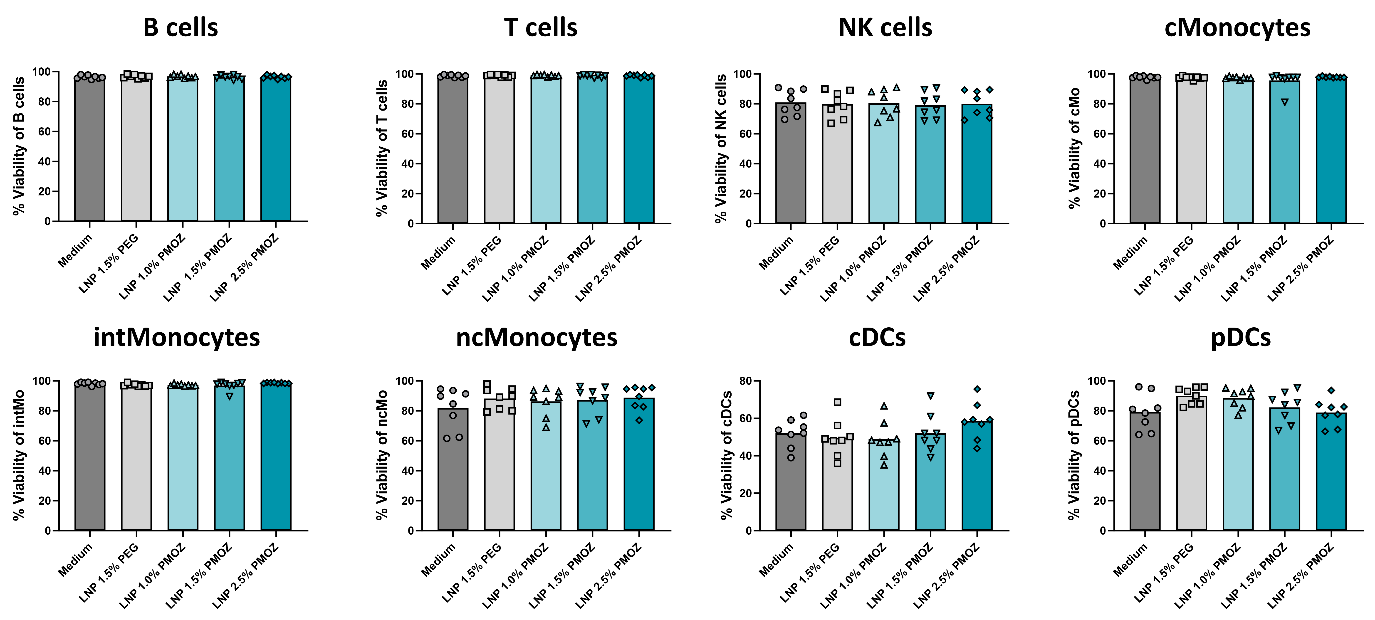


Supplementary Figure 3: Viability of PBMCs treated with LNPs that encapsulate eGFP mRNA. FVS780-positive cells (dead cells) were identified by flow cytometry. Statistical analysis was performed with a one-way ANOVA with Tukey’s multiple comparison test. *P < 0.1, **P < 0.01, ***P <0.001, ****P < 0.0001.


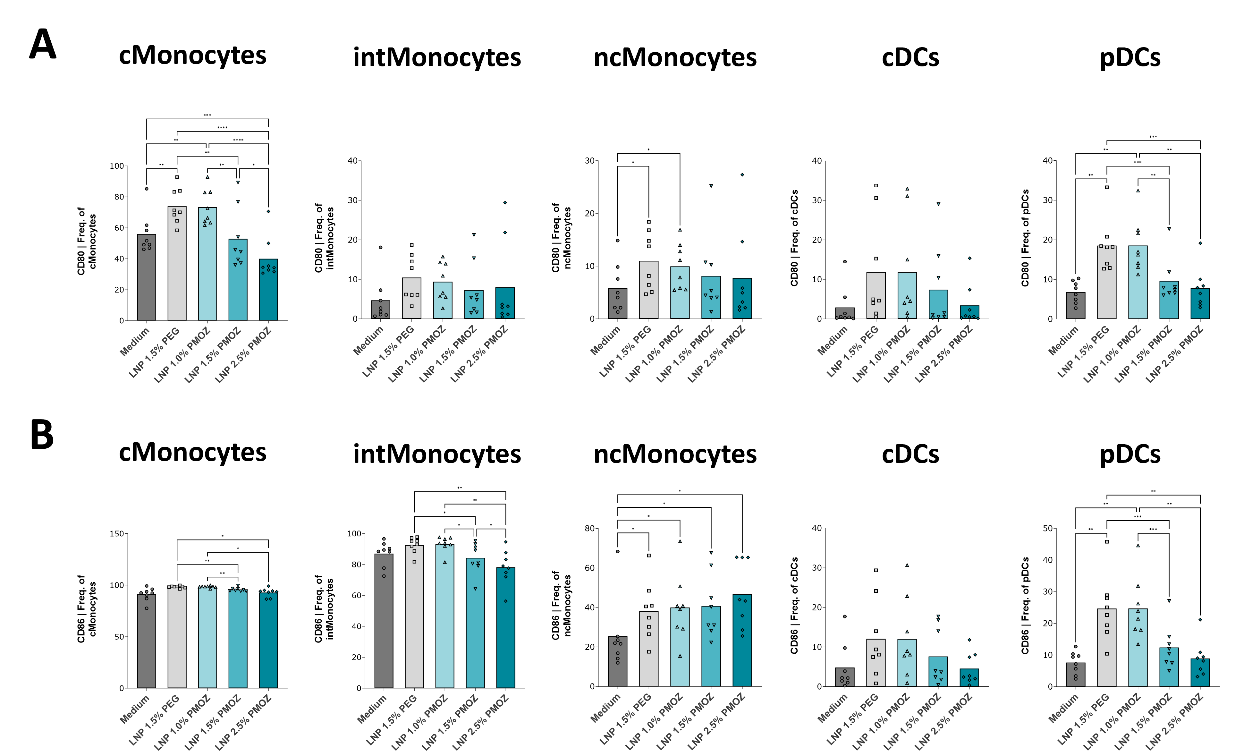


Supplementary Figure 4: Analysis on innate immune cell activation through the surface expression of activation markers CD80 (A) and CD86 (B). Percentages are given and bars represent the mean of 8 donors. Statistical analysis was performed with a Brown-Forsythe and Welch ANOVA with Dunnett’s T3 multiple comparison test. *P < 0.1, **P < 0.01, ***P <0.001, ****P < 0.0001.


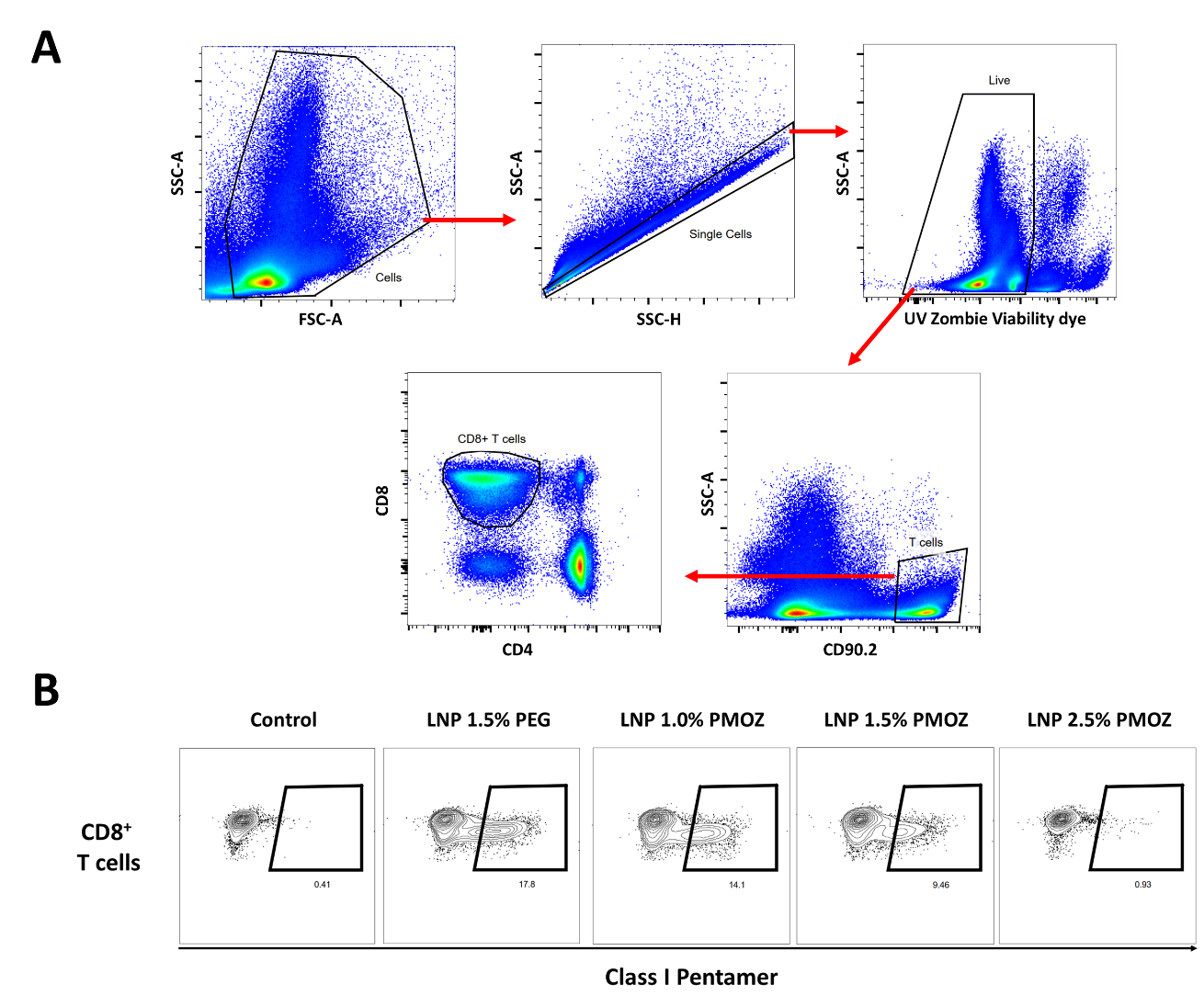


Supplementary Figure 5: Analysis of antigen-specific CD8^+^ T cells in mouse spleens on day 28. The gating strategy employed (A) and representative contour plots of CD8^+^ class I pentamer^+^ cells (B) are shown.


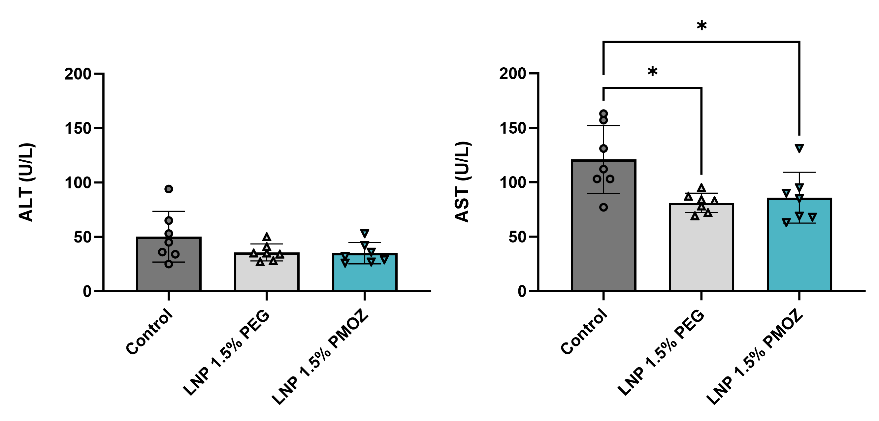


Supplementary Figure 6: ALT and AST levels in the sera of animals treated with buffer (control), LNP 1.5% PEG and LNP 1.5% PMOZ. Sera were collected with 14 hours after the primary vaccination. Statistical analysis was performed with a one-way ANOVA with Tukey’s multiple comparison test. *P < 0.1, **P < 0.01, ***P <0.001, ****P < 0.0001.
